# Meta-analysis of glioblastoma multiforme *versus *anaplastic astrocytoma identifies robust gene markers

**DOI:** 10.1186/1476-4598-8-71

**Published:** 2009-09-04

**Authors:** Jonathan M Dreyfuss, Mark D Johnson, Peter J Park

**Affiliations:** 1 Partners HealthCare Center for Personalized Genetic Medicine, Brigham and Women's Hospital, Boston, MA 02115, USA; 2 Department of Neurosurgery, Brigham and Women's Hospital, Boston, MA 02115, USA; 3 Center for Biomedical Informatics, Harvard Medical School, Boston, MA 02115, USA; 4 Children's Hospital Informatics Program, Boston, MA 02115, USA

## Abstract

**Background:**

Anaplastic astrocytoma (AA) and its more aggressive counterpart, glioblastoma multiforme (GBM), are the most common intrinsic brain tumors in adults and are almost universally fatal. A deeper understanding of the molecular relationship of these tumor types is necessary to derive insights into the diagnosis, prognosis, and treatment of gliomas. Although genomewide profiling of expression levels with microarrays can be used to identify differentially expressed genes between these tumor types, comparative studies so far have resulted in gene lists that show little overlap.

**Results:**

To achieve a more accurate and stable list of the differentially expressed genes and pathways between primary GBM and AA, we performed a meta-analysis using publicly available genome-scale mRNA data sets. There were four data sets with sufficiently large sample sizes of both GBMs and AAs, all of which coincidentally used human U133 platforms from Affymetrix, allowing for easier and more precise integration of data. After scoring genes and pathways within each data set, we combined the statistics across studies using the nonparametric rank sum method to identify the features that differentiate GBMs and AAs. We found >900 statistically significant probe sets after correction for multiple testing from the >22,000 tested. We also used the rank sum approach to select >20 significant Biocarta pathways after correction for multiple testing out of >175 pathways examined. The most significant pathway was the hypoxia-inducible factor (HIF) pathway. Our analysis suggests that many of the most statistically significant genes work together in a *HIF1A*/*VEGF*-regulated network to increase angiogenesis and invasion in GBM when compared to AA.

**Conclusion:**

We have performed a meta-analysis of genome-scale mRNA expression data for 289 human malignant gliomas and have identified a list of >900 probe sets and >20 pathways that are significantly different between GBM and AA. These feature lists could be utilized to aid in diagnosis, prognosis, and grade reduction of high-grade gliomas and to identify genes that were not previously suspected of playing an important role in glioma biology. More generally, this approach suggests that combined analysis of existing data sets can reveal new insights and that the large amount of publicly available cancer data sets should be further utilized in a similar manner.

## Background

High-grade gliomas, which include World Health Organization grade III astrocytomas (anaplastic astrocytoma: AA) and grade IV astrocytomas (glioblastoma multiforme: GBM), are the most common intrinsic brain tumors in adults and are almost universally fatal. GBMs are particularly invasive and aggressive. Patients diagnosed with GBM have a median survival time of one year [1], and less than 20% survive two years [2]; in contrast, the median survival for patients with AA is 30 months [1]. Nearly all GBMs (>90%) are primary, i.e. they develop *de novo *with no evidence of a less malignant precursor lesion, whereas secondary GBMs develop from lower-grade astrocytomas [3]. Histological criteria are currently the basis for tumor grading and prognosis, with GBM showing increased necrosis, vascular proliferation, nuclear pleomorphism, mitoses and invasiveness when compared to AA. The molecular basis for the histological and prognostic differences between grade III and grade IV astrocytomas remains an area of active investigation, e.g. one study found genes associated with necrosis in high-grade gliomas [4]. A deeper understanding of the basis for these differences may lead to new therapeutic strategies for treating these tumors.

Differences in chromosomal alterations in AA and GBM have been described in several studies. For example, loss of heterozygosity for chromosome 10 was often observed in high-grade astrocytomas, and its frequency was found to be different between AA and GBM [5]. Aberrations involving *p53, EGFR, PTEN*, and other genes have also been reported as having different frequencies in AA and GBM. Importantly, differences within the same grade were also observed. Aberrations on chromosome 10, for example, were found to be an independent, adverse prognostic marker for survival, even after accounting for age and grade [5,6]. With the advent of microarrays, molecular portraits of these tumor grades were refined, and expression profiling was found to be a better predictor of outcome than histological criteria [7,8]. These and other studies revealed the presence of molecular subgroups of malignant gliomas. One recent study identified three molecular subclasses of GBM that were characterized by proneural, proliferative, and mesenchymal mRNA expression signatures [9], and another isolated an expression signature that distinguished survival phenotypes [10].

Although a number of expression profiling studies have been performed on AA and GBM, they give conflicting results with regard to the list of relevant, differentially expressed genes between GBM and AA. This variability may be due to several factors. Most importantly, the sample sizes for these studies were relatively small due to the limited availability of suitable specimens and the significant costs associated with these studies. Other factors include differences in: the quality of the tissue specimens used (e.g. presence of non-tumor brain tissue or extensive necrosis), the microarray platforms used, the statistical methods employed to identify differentially expressed genes [11], and patient demographics such as age, gender, and race. Given the large number of factors that influence the list of differentially expressed genes, it is not surprising that gene lists from independent studies show little overlap. This lack of overlap has been observed in nearly all diseases in which microarrays have been employed, although the extent of the discrepancy depends on the heterogeneity of the disease [12].

To compile the most accurate and robust list of relevant genes, we performed a meta-analysis of multiple independent publicly available data sets, mostly from the Gene Expression Omnibus (GEO). GEO is the largest public repository of microarray data; it now contains over 250,000 samples and its size is rapidly increasing [13]. While many of the published microarray data sets are centrally stored through GEO, the format and quality of the data sets are variable, and the annotations, both in terms of the probes on the platform and the sample phenotypes, are often incomplete. Thus, integrating information from these data sets requires significant bioinformatics analysis. In this study, many data sets were examined, and four data sets that satisfied our criteria for suitability in meta-analysis were selected. The resulting list of genes that are differentially expressed between AA and GBM is likely to be more robust and stable than that derived from any individual study to date.

## Results

### Identification of appropriate data sets

To identify gene expression differences between AA and GBM, we searched GEO and many other databases containing publicly-available microarray data for data sets that contain information for both grades. One possible strategy for meta-analysis would have been to collect all data sets containing GBMs and all data sets containing AAs separately, and to then perform a single differential analysis. However, this could potentially lead to artifactual results due to methodological or technical differences among the studies, as mentioned above. Platform differences, for example, can have a significant influence on the results of microarray analyses. We have previously shown that even the differences arising from the use of successive generations of microarray platforms produced by the same company (e.g. Affymetrix) can be larger than the differences among patient samples [14]. While such artifactual effects can be reduced somewhat with careful normalization and use of robust statistics, they cannot be eliminated. A more conservative approach is to combine the information obtained at the level of "within-experiment" gene lists, so that platform-specific and other biases are reduced.

To increase the reliability of the results, we further enforced stringent criteria for data inclusion. Distinct expression profiles exist between primary and secondary GBMs [15], and it is estimated that 95% of GBMs are primary [3]. Hence, to pinpoint targets for forced grade reduction of primary GBMs, we focus exclusively on contrasting AAs to primary GBMs. (Although there is not sufficient data for a meta-analysis of AAs *versus *secondary GBMs, interesting findings have come out of this comparison, e.g. [16].) We found four large *in vivo *expression data sets, three from GEO and one from UCLA, that assay AAs and primary GBMs. All four studies used the Affymetrix (Santa Clara, CA) human U133 platform. This similarity of expression platforms simplifies the analysis, although the same process with minor modifications would have worked well even with differing platforms. Table 1 summarizes the data used for meta-analysis. Note that this platform consistency is not by design; our search of human *in vivo *expression studies did not yield any other studies that assayed five or more GBMs and AAs in patient samples, regardless of platform.

**Table 1:** Summary of the data sets

	**Petalidis**	**Phillips**	**Sun**	**Tso**	**TOTAL**
AA	19	21	19	9	68
GBM	39	56	81	45	221
TOTAL	58	77	100	54	289
GEO ID	GSE1993	GSE4271	GSE4290	(at UCLA)	
Affy chip	U133A	U133A and U133B	U133 plus 2.0	U133A	
Journal	Mol Cancer Ther	Cancer Cell	Cancer Cell	Cancer Research	
Year	2008	2006	2006	2006	

### Statistical approach to combining data sets

Analysis of differential expression in a single data set has been examined in great detail in the past decade [17,18]. See [11] for a comparison of some commonly used methods. For this work, we focus on the methods for combining multiple data sets, i.e. for combining the scores of individual features across microarray experiments. Two classical statistical techniques that combine a feature's p-values directly are Fisher's method [19,20], which relies on the sum of the logarithm of the p-values, and an alternative method proposed by Stouffer et al. (1949; cited in [21]), which transforms p-values into z-scores. Fisher's method was used, for instance, for analysis of microarray data on breast cancer [22], and both Fisher's and a weighted version of Stouffer's method were applied to study prostate cancer [23,24]. Meta-analytic methods have also been developed specifically for genomics, many of which rely on traditional statistical approaches such as random effects [25,26] and Bayesian modelling [27,28], and some techniques have been advanced specifically for combining cancer microarray data [29]. Meta-analysis for genomics has accrued so much literature that there is now a book dedicated to the topic [30].

Breitling et al. (2004) and Hong and Breitling (2007) have proposed a simple, intuitive method that evaluates genes based only on the product (or the sum) of its ranks [31,32]. This method ranks each feature (such as a gene) within an experiment based on that feature's score (say, a t-statistic), and then combines these ranks, rather than combining the data or p-values themselves. For example, if a certain gene is the most differentially expressed gene in one experiment and is the tenth most differentially expressed gene in the three others, then its rank sum will be 1+10+10+10 = 31 and its rank product will be 1*10*10*10 = 1000, where the smaller is the rank sum or rank product, the more significant is the gene. The two approaches differ only in how they penalize the larger ranks; the rank product becomes very large even with a single high rank. Because rank-based procedures do not make assumptions about the model and parameters from which the data came, they are termed *non-parametric*.

We chose to use a rank-based method because: 1) in practice, the main purpose of microarray experiments is to rank genes rather than to obtain precise estimates of their statistical significance, since the number of statistically significant genes often greatly exceeds the number of genes that can be validated [33], 2) non-parametric analyses are more robust in general, 3) the techniques and assumptions used in the estimation of p-values and the subsequent correction for multiple hypothesis testing may be different between data sets and may not be directly comparable, and 4) using non-parametric methods to rank genes has proven highly effective in the context of genomics. Although more sophisticated rank-based procedures are available [34], the rank sum and rank product methods have been shown to give good results on microarray data [32]. Because the rank sum technique is more robust than the rank product approach and is preferable when the variance of some features may be larger than others [35], we employ the rank sum procedure.

As a complement to the ordered gene list for each study, which we derive using moderated t-statistics [36], we also quantify differentially activated pathways between GBM and AA. The benefit of testing the significance of *a priori *defined gene sets (which correspond to pathways in this article) is that the recognition of such pathways may allow for better elucidation of the underlying biology, improved drug target development, and greater generalizability [37]. In this work, we used a statistical method that we previously developed to identify significant gene sets while accounting for the differing sizes of gene sets and their correlation structure [38].

### Meta-analysis gene list

**Figure 1:**
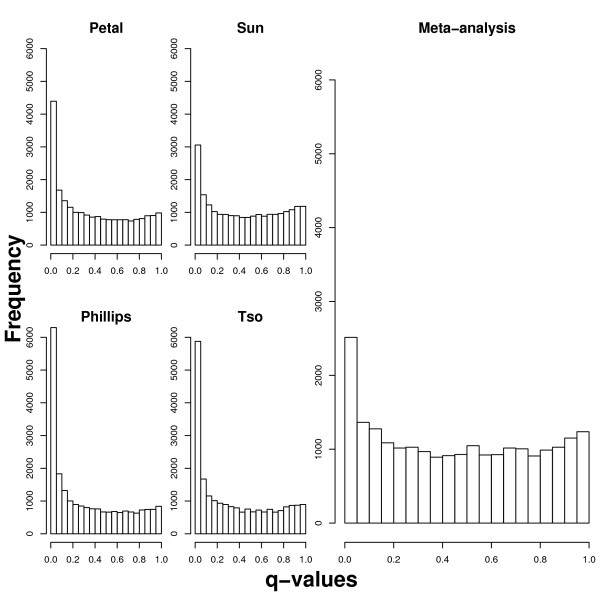
**Gene q-value histograms**. Histograms depicting the significance levels of probe sets from each of the four studies, and from the meta-analysis.

Of our 22,215 probe sets (see *Methods*), we identified 933 with rank sum based q-values [39], an analogue of p-values when many features are being tested, below 1%. These meta-analytic statistics provide a combined ranking of significant genes while not allowing strong p-values from any individual study to dominate the results. The amount of differential expression observed is illustrated in Figure 1, which shows histograms depicting the q-values from each study on the left and the relatively conservative q-values from the meta-analysis on the right. The high proportion of genes with low q-values indicates that many more genes are found to be differentially expressed than expected by chance.

**Figure 2:**
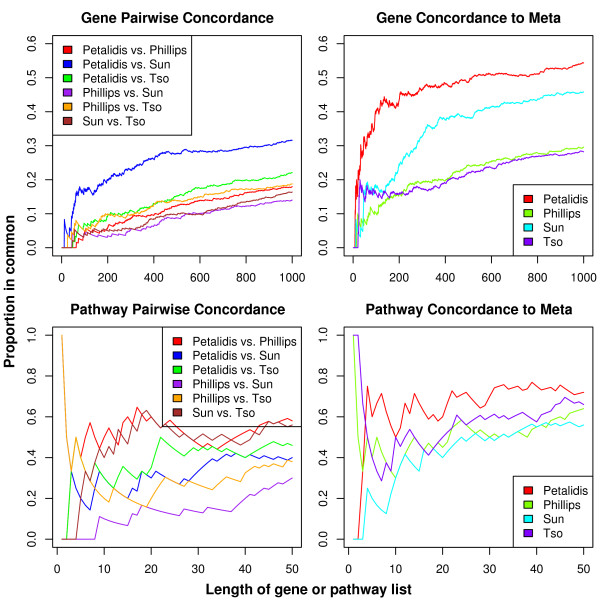
**CAT plots**. Concordance At the Top plots comparing ranked probe set lists from pairs of studies (top left) and from each study to the meta-analysis probe set list (top right), and similarly for pathways (bottom row).

The top row of Figure 2 displays these findings through Concordance At the Top (CAT) plots [40], which quantify the concordance of two lists along list ranks. These plots have a straightforward interpretation. For example, if two gene lists share in common 80 of their top 100 genes, then at rank 100 their concordance would be 80%. Hence, the gene plots in Figure 2 show that no study dominates the final meta-analysis gene list and that these studies' gene lists, although far more concordant than would be expected by chance, contain ample heterogeneity. This coupling of apparent heterogeneity with abundant meta-analytic significance points to a wealth of information only available through a powerful meta-analysis.

Information on the top 30 meta-analysis genes is displayed in Table 2, while Additional file 1 contains information for all probe sets. A survey of the literature indicates that several of these top 30 genes have been demonstrated to play an important role in the biology of malignant gliomas. These include *CHI3L1 *[41,42], *FN1 *[43,44], *CLIC1 *[45], *VEGFA *[46], *IGFBP2 *[47], *ADM *[48], and *COL4A1/COL4A2 *[49].

**Table 2:** Comparison of top genes

**Petalidis**	**Phillips**	**Sun**	**Tso**	**Meta**	**Meta Gene Name**
TIMP1*	CALR*	SRPX2	RPS3A*	GLUD1*	glutamate dehydrogenase 1
ADCY2	CSF1*	ADAM12*	SC5DL	GLUD2	glutamate dehydrogenase 2
LAMC1*	MTSS1*	ALDH5A1*	ZNF192*	CLIC1*	chloride intracellular channel 1
ABHD6	ANXA2P3	LAMB1*	ANXA1*	COL4A1*	collagen, type IV, alpha 1
COL5A2	CRHR2*	ACTN1*	MYST4	CCDC109B	coiled-coil domain containing 109B
**CSDC2**	BMP8A*	NTSR2*	CAPG*	VEGFA*	vascular endothelial growth factor A
RBP1*	CALCRL	FAM69A*	ACVR2B*	COL4A2*	collagen, type IV, alpha 2
EFEMP2*	CG030*	PPP2R5A*	SPP1*	RASL10A	RAS-like, family 10, member A
COL4A1*	SCD*	HRSP12*	CHI3L1*	IGFBP2*	insulin-like growth factor binding protein 2, 36kDa
KIAA049	SORBS1	COL5A2	MYOZ2	FN1*	fibronectin 1
IGFBP2*	PEX5	KDELR3	SLC25A21	PPP2R5A*	protein phosphatase 2, regulatory subunit B', alpha isoform
LGALS1*	ADCY2	SERPINH1*	TMLHE	PSRC1*	proline/serine-rich coiled-coil 1
PLP2	**GLUD2**	SRF*	GNAL	LUZP2	leucine zipper protein 2
CLIC1*	EDG8	ZHX2	AKT3*	LPIN1	lipin 1
USH1C*	HSDL2	LOC727942	TSC22D2	ARL4C	ADP-ribosylation factor-like 4C
FKBP9	PI4K2A	FOSL2*	RICS*	PDIA4*	protein disulfide isomerase family A, member 4
BMP2*	GLUD1*	LOXL2*	**LUZP2**	ADM*	adrenomedullin
AKAP6	DCAKD	CHPF	NET1*	CSDC2	cold shock domain containing C2, RNA binding
LDHA*	CCL19	LMNA*	GUSB*	TMSB10*	thymosin, beta 10
DHTKD1	RAB40C	SLC16A3	C11orf41	CHI3L1*	chitinase 3-like 1 (cartilage glycoprotein-39)
ADM*	DIP2C	BICD1	GLUD1*	NET1*	neuroepithelial cell transforming gene 1
KIAA0746	LOC645226*	IQCK	LITAF*	MCAM*	melanoma cell adhesion molecule
KDELR2*	TNKS2*	FAM129A*	CEP350*	LDHA*	lactate dehydrogenase A
LBH	AP2B1*	COL4A2*	**RASL10A**	COL1A2*	collagen, type I, alpha 2
COL4A2*	HIRA	CSGlcA-T	HLA-C*	ALDH2*	aldehyde dehydrogenase 2 family (mitochondrial)
CALD1*	MAP2K3*	TGFB1I1*	CLCA2*	COL3A1	collagen, type III, alpha 1 (Ehlers-Danlos syndrome type IV, autosomal dominant)
**COL3A1**	GRWD1	C21orf7	S100A11*	LAMC1*	laminin, gamma 1 (formerly LAMB2)
TMED9	**LPIN1**	**ARL4C**	PLCB1*	SLC1A4*	solute carrier family 1 (glutamate/neutral amino acid transporter), member 4
TAGLN2*	VPS13D*	ZGPAT	TRIP4*	MSN*	moesin
PELO	MUC8*	PVR*	SERPINA3*	CNTN1*	contactin 1

The first 5 columns contain the top 30 genes from each study and from the meta-analysis. Column 6 contains gene names for the top meta-analysis genes, all of which have a q-value < 0.001. Genes in the first 4 columns that appear in the top 30 of the meta-analysis gene list are in bold, while those that are related to the PubMed query "glioma OR cancer OR astrocytoma" are given an asterisk.

### Comparison to literature

An automated search of the PubMed database for relevant abstracts related to the top 30 genes derived from each study and from the meta-analysis (using the search term "glioma OR cancer OR astrocytoma") indicates that the meta-analysis genes are associated with the greatest number of relevant citations and contain the highest proportion of genes with relevant citations (see Table 3). For example, the gene *VEGFA*, which is known to be important in glioma and generates more than 750 pertinent citations on its own, is 6^th ^in the meta-analysis list but does not fall among the top 30 genes on any of the individual studies' lists.

**Table 3:** Counts of relevant citations

	**Petalidis**	**Phillips**	**Sun**	**Tso**	**Meta**
# citations (PubMed)	128	50	58	127	154
% cited (PubMed)	53%	47%	57%	67%	73%
% cited (Ingenuity)	47%	33%	27%	40%	60%

Comparison of relevant citation counts of top 30 genes from each of the four studies and from the meta-analysis.

The largest number of relevant citations derived from the top 30 genes of any single study is 128. To ensure that *VEGFA *does not dominate the comparison, we assigned it the same number of citations as the second best performing gene from all of Table 2 (which is *SPP1*, with 41 relevant citations). After this citation reduction for *VEGFA*, the meta-analysis list still generates 154 glioma/cancer/astrocytoma-related citations. The meta-analysis list's top 30 genes also have more citations related to the search term (22 genes) than any of the four studies. We further substantiated the results obtained from PubMed by evaluating these same gene lists using the manually curated Ingenuity Pathways Analysis software program (Ingenuity Systems, ). Ingenuity found that 60% of the top 30 unique genes from the meta-analysis have known connections to cancer, which is a 13% increase above the top-performing individual study.

### Meta-analysis pathway list

We next performed pathway analysis to determine whether the genes identified by the meta-analysis might work cooperatively. Of 178 Biocarta  pathways, 21 had a q-value below 2%. These 21 gene sets are shown in Table 4, while Additional file 2 contains the information for all gene sets. These significance results coupled with the bottom row of Figure 2 show that, similarly to our gene-level analysis, we are able to glean insight from heterogeneous pathway lists using the rank sum method. As hoped, owing to the greater reproducibility in general of gene set analyses, the pathway lists of the individual studies also exhibit greater concordance to each other than do their gene lists (this can be witnessed by examining fixed percentiles of the gene and pathway lists in Figure 2.)

**Table 4:** Top pathways

	**Pathway**	**Size**	**% up**	**Change**	**q-value**
HIF	Hypoxia-Inducible Factor in the Cardiovascular System	31	65	up	0
PROTEASOME	Proteasome Complex	37	89	up	5.00E-04
MYOSIN	PKC-catalyzed phosphorylation of inhibitory phosphoprotein of myosin phosphatase	25	28	down	0.00367
VITCB	Vitamin C in the Brain	28	61	up	0.0055
LYMPHOCYTE	Adhesion Molecules on Lymphocyte	27	81	up	0.00942
NOS1	Nitric Oxide Signaling	53	25	down	0.00942
P53HYPOXIA	Hypoxia and p53 in the Cardiovascular system	36	72	up	0.00942
SALMONELLA	How does salmonella hijack a cell	26	81	up	0.00942
ATM	ATM Signaling	36	72	up	0.00942
CASPASE	Caspase Cascade in Apoptosis	43	72	up	0.00942
PAR1	Thrombin signaling and protease-activated receptors	39	31	down	0.00942
G2	Cell Cycle: G2/M Checkpoint	43	70	up	0.00942
TSP1	TSP-1 Induced Apoptosis in Microvascular Endothelial Cell	21	86	up	0.00946
ACTINY	Y branching of actin filaments	31	77	up	0.0109
NEUTROPHIL	Neutrophil and Its Surface Molecules	21	81	up	0.0152
ATRBRCA	Role of BRCA1, BRCA2 and ATR in Cancer Susceptibility	40	85	up	0.0152
MONOCYTE	Monocyte and its Surface Molecules	30	80	up	0.0155
FAS	FAS signaling (CD95)	65	62	up	0.0177
PGC1A	Regulation of PGC-1a	54	24	down	0.0184
CELLCYCLE	Cyclins and Cell Cycle Regulation	37	73	up	0.0186
VEGF	VEGF, Hypoxia, and Angiogenesis	54	61	up	0.0186

Pathways with overall q-value < 2%. Columns are the full Biocarta pathway name, the number of genes in the pathway, the percentage of genes that are expressed more highly in GBM relative to AA, the direction of change of the pathway in GBM vs. AA, and the q-value.

**Figure 3:**
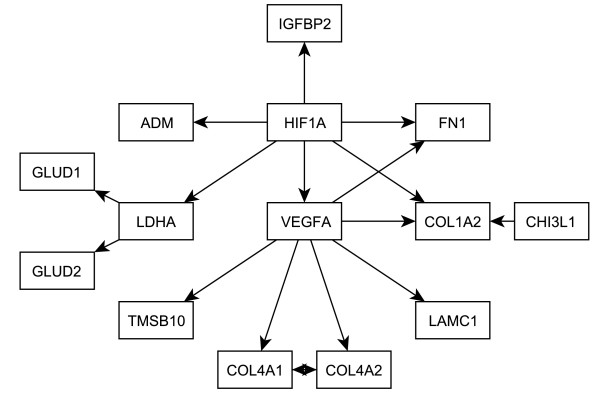
**Relationship of *HIF1A *and *VEGFA *to top meta-analysis genes**. Relationships from the literature among *HIF1A*, *VEGFA*, and some of the top 30 meta-analysis genes.

Several themes emerged from the pathway analysis. The most statistically significant gene set identified in the meta-analysis was the hypoxia-inducible factor (HIF) pathway, which has been repeatedly implicated in GBM. This pathway of 31 genes was highly upregulated in GBM and performed extremely well in all four studies, ranking #1 twice, #3, and #9. The *HIF1A *gene encodes a transcription factor that is induced by hypoxia and that controls the expression of a set of genes that promote angiogenesis and invasion. Importantly, several of the top 30 genes on the meta-analysis gene list are direct *HIF1A *transcriptional targets, including *VEGFA *[50,51], *ADM *[52], *IGFBP2 *[53], *LDHA *[54], and *FN1 *[55]. *VEGF*, in turn, induces expression of a number of collage subtypes and extracellular matrix proteins needed for the generation of new blood vessels and for invasion [56]. Among these are several additional genes listed among the top 30 on the meta-analysis list, including *LAMC, COL4A1, COL4A2, COL1A2 *and *FN1*. *TMSB10 *can also be regulated by *VEGF *[57]. When considered together, these genes suggest differential activation of the HIF1A/VEGF network in GBM when compared to AA. Figure 3 illustrates the interrelationship between *HIF1A*, *VEGFA *(whose pathway is ranked 21^st^), and related genes that are found among the top 30 on the meta-analysis list.

## Discussion

Because it is not feasible to control for all the factors influencing gene expression in studies of human tumor specimens, it is important to aggregate as much high-quality data as possible to eliminate these sources of bias. Given the large volume of microarray data being generated by laboratories across the world, taking advantage of these data through meta-analysis has become a fruitful and inexpensive yet under-utilized approach. In our comparison of AA and GBM, including only four, albeit very large, studies does leave our results somewhat dependent on the quality of these microarray data sets. However, our methodology disfavors genes whose top ranks are not consistent. As future high-quality data sets become available, they can be incorporated into this framework to validate and improve the stability and accuracy of these results without the worry that such additions will lead to dramatic alterations in the ordering of features. Such benefits offer practical, concrete reasons for our choice of meta-analytic methodology and provide promising evidence for applying this analysis workflow to other pressing conditions.

Other similar studies may further benefit from using survival time as the phenotype of interest. Molecular signatures have been found to be better predictors of survival than histological grade in some cases [7,8], i.e. the survival associated with tumors whose molecular profile was an "exception" to their histological grade was more strongly dictated by gene expression profile than by grade. However, Petalidis et al. [58] demonstrated that molecular signatures derived from histological grading of gliomas can be robust prognostic indicators whose accuracy in delineating survival subclasses may outperform classifiers trained on survival data, histological grade *per se*, and tumor subtypes defined by other studies. Nonetheless, meta-analyses that exploit survival data could potentially provide a more relevant list of candidate genes and a more powerful molecular classification of tumors. In this study, we had to restrict our focus to tumor grade because not all of our data sets had survival data available. This underscores the need for careful clinical annotation of the samples in these studies, even if a study does not involve survival analysis.

Although there is already a deep literature on the molecular properties of glioblastoma multiforme, The Cancer Genome Atlas project (TCGA) chose GBM as its first cancer to study [59]. TCGA is an ongoing effort coordinated by the NIH in which numerous groups from many institutions collaboratively utilize the gamut of genome analysis technologies to accelerate our understanding of the molecular basis of cancer . It will be important to integrate our findings with those of TCGA (which compares GBM to normal tissue) and to identify pathways that are differentially expressed between GBM and AA with the hope of targeting these pathways therapeutically and increasing the survival of patients with GBM so that it approaches that of AA. Evidence that this approach is a useful one can be found in the fact that experimental and clinical studies have shown that agents that target the HIF1A/VEGF network can decrease tumor growth and prolong survival in both animals and humans [60,61].

## Conclusion

We have identified >900 probe sets and >20 pathways whose expression is statistically significantly different between GBM and AA. These feature lists are likely to be more accurate and stable because of the greater sensitivity and specificity that result from integration of data. Further, both the top genes and pathways implicate HIF1A/VEGF network activation as a major contributor to the increased growth and invasion displayed by GBM when compared to AA. The importance of these pathways is also evidenced by the utility of VEGF and HIF1A inhibitors in decreasing glioma growth and prolonging survival *in vivo*. This type of meta-analysis could be utilized to aid in the diagnosis and prognosis of malignant gliomas, and in the development of new therapies for these devastating tumors.

## Methods

### Data description and processing

Both the Human Genome U133A and U133B Affymetrix platforms contain >22,000 probe sets with no overlap between these two arrays. The Human Genome U133 Plus 2.0 array is composed of all of the probe sets on each of these two arrays as well as 9,921 new probe sets, giving it >54,000 probe sets in total. To accommodate all four of our studies, we used the 22,215 probe sets from the U133A array. Note that these are one-channel microarrays, so that only one sample is hybridized to each microarray. Hence, no controls were involved in our direct comparison of AAs to GBMs and there is no bias due to different controls used.

Petalidis et al. (2008) identified molecular signatures from primary human astrocytic tumors that define survival prognostic subclasses [58]. Phillips et al. (2006) determined molecular subclasses of human gliomas useful in prediction of prognosis and disease progression [9]. Sun et al. (2006) examined stem cell factor in primary human gliomas [62]. Tso et al. (2006) identified glioblastoma associated genes in primary and secondary human gliomas [15] and deposited the data at UCLA: . Although the data set of Freije et al. [7] would have satisfied our criteria, its samples heavily overlap with those of Tso et al. [15].

Only the studies of Phillips et al. [9] and Tso et al. [15] had raw data (Affymetrix CEL) files available. We preprocessed these using RMA normalization [63] from the *affy *package [64], which is the same method employed by Petalidis et al. [58]. Sun et al. [62] already applied the normalization procedure of Li and Wong [65]. These differences in normalization technique, however, do not pose a hazard to this analysis due to our combination of "within-experiment" feature lists. All data was put on a log base 2 scale.

### Statistical analysis

The implementation of the meta-analysis followed several steps. Firstly, features (either probe sets or pathways) were scored within each study. Secondly, features were ranked within each study by the magnitude (i.e. absolute value) of their respective statistic (say, a t-statistic), where the statistic closest to zero was given rank one, while that furthest from zero received the largest rank. Negative signs were then given to ranks corresponding to negative statistics to allow for asymmetric (i.e. if there are more upregulated than downregulated features, or *vice versa*) feature lists. Thirdly, a feature's ranks were summed across the four studies, assigning each feature a single rank sum. Lastly, these rank sums were compared to null ranks sums, derived by randomly permuting column labels and re-running the analysis, to obtain q-values. Note that according to this method, rank sums with larger magnitude are more significant.

To create per-study gene lists, we employed the empirical Bayes package *limma *[66], which offers a moderated t-statistic [36] for each gene, along with its associated p-value and conservatively estimated [67] q-value. The empirical Bayes methodology, and this package in particular, have been found in independent bioinformatics comparisons to be highly robust [11] and a preferred analysis method for Affymetrix GeneChips [68]. Annotation was derived from the Affymetrix HG-U133A annotation files in CSV format, downloaded from NetAffx Analysis Center .

Gene sets were derived from the Gene Set Enrichment Analysis (GSEA) Molecular Signature Database v2.5 [69], where we extracted Biocarta pathways from the "C2: curated gene sets" collection that hold between 20 and 500 genes. This gave 178 Biocarta pathways. Gene set elements were converted from gene symbols to U133A probe sets using GSEA's *chip2chip *tool [37,69]. Analysis of gene sets was performed using our *SigPathway *Bioconductor package [38], which compares each gene set to a column and row permutation null distribution separately, giving two normalized enrichment scores per gene set. These enrichment scores were used separately for the column permutation and row permutation q-values in Additional file 2. Otherwise, within-experiment gene set rank was computed using the minimum (in magnitude) of these two scores for the overall q-value.

To compare the gene lists to the literature, we were able to automate our search of relevant citation counts for top genes by using the *hgu133a *package, which maps Affymetrix probe sets to Entrez Gene identifiers to PubMed identifiers, and the *annotate *package, which allows searching of PubMed abstracts. All statistical analysis was done in the R software [70] using packages from the Bioconductor project [71].

## Competing interests

The authors declare that they have no competing interests.

## Authors' contributions

PJP designed the study, JMD carried out the study, MDJ provided help with biological interpretation, and JMD, MDJ, PJP wrote the paper. All authors read and approved the final manuscript.

## Supplementary Material

Additional file 1 **Probe set table**. This table contains analysis results for all probe sets from the human U133A array. The columns contain the gene symbol, the gene name, the average fold change of GBM vs. AA, the rank sum, the q-value derived from this rank sum, and the gene's ranks in all four studies (where the ranks were multiplied by -1 when their corresponding moderated t-statistic was negative). Click here for file

Additional file 2 **Gene set table**. This table contains analysis results for all 178 Biocarta pathways. The columns contain the number of genes in the pathway, the direction of change with respect to GBM vs. AA, the percentage of genes that are up in GBM relative to AA, the overall q-value, the q-values from the column and row permutations of *SigPathway*, and the rank sum. Click here for file
